# Meta-analysis of the changes of peripheral blood regulatory T cell to CD4^+^ T cell ratio in patients with systemic sclerosis

**DOI:** 10.1038/srep43532

**Published:** 2017-03-02

**Authors:** Chuiwen Deng, Wenli Li, Si Chen, Yongzhe Li

**Affiliations:** 1 Department of Rheumatology and Clinical Immunology, Peking Union Medical College Hospital, Chinese Academy of Medical Sciences & Peking Union Medical College, Key Laboratory of Rheumatology and Clinical Immunology, Ministry of Education, 100073, Beijing, China; 2 Department of Rheumatology, China-Japan Friendship Hospital, Yinghua East Road, Chaoyang District, 100029, Beijing, China; 3 Department of Clinical Laboratory, Beijing Anzhen Hospital, Capital Medical University, 2# Anzhen Road, Chaoyang District, 100029, Beijing, China

## Abstract

Current reports on the changes in peripheral blood regulatory T cell (Tregs) to CD4^+^ T cell ratio in systemic sclerosis (SSc) patients are varied in their conclusions. We therefore performed a meta-analysis to identify the actual change in the proportion of peripheral Tregs in SSc. Three databases, namely EMBASE, ISI web of knowledge, and Pubmed were systematically searched for relevant literature. Approximately 250 SSc patients and controls from several studies were included in this analysis. Comprehensive Meta Analysis Version 2.0 software was used to conduct the meta-analysis. Six studies were included in the meta-analysis. Results of the meta-analysis showed high degree of heterogeneity (*I*^*2*^ = 96.98), and a random-effect model was used in the subsequent analysis. The ratio of circulating Tregs to CD4^+^ T cell in SSc was lower than in controls, but not statistically significantly so (−0.61 ± 0.94, P = 0.52). Subgroup analysis did not identify any potential source of heterogeneity. This meta-analysis indicated that Tregs might play a less prominent immunosuppressive role in the immune system in SSc patients, but needs further confirmation.

Systemic sclerosis (SSc) is a heterogeneous autoimmune disease characterized by chronic inflammation leading to fibrosis of the skin and organs and is often related to vasculopathy and immunologic abnormalities^1^. The pathogenesis of SSc is still unclear, but clues found in clinical practice may provide some details in this regard.

Immune dysfunction is one of the most important clinical symptoms of SSc and has been comprehensively studied. Increasingly, research in the area has confirmed that abnormalities of the innate and adaptive immune systems in SSc patients result in the production of auto-antibodies and the activation of cell-mediated autoimmunity^2^. As one of the major mononuclear cells that infiltrate skin lesions^3^, T cells play a vital role in modulating cell-mediated autoimmunity. There have been reports showing that infiltrated T cells are activated, show oligoclonal expansion, and persist in lesion skins of SSc patients for a long time^4^, all of which support the view that these cells participate in the pathogenesis of the disease. Therefore, answering how the balance of T cells in affected may help elucidate the mechanisms underlying the pathogenesis of SSc.

In recent years, mounting evidence suggests that T regulatory cells (Tregs) play a key role in maintenance of self-tolerance and modulation of autoimmune responses by controlling autoreactive T cells^5^, even though Tregs represent only about 5% of the peripheral CD4^+^ T cells in humans. To explore the relationship between Tregs and the other T cells in SSc patients, studies have been performed to explore changes in the ratio of circulating Tregs to CD4^+^ T cell and the functional consequences of these changes.

A few reports from groups have specifically addressed this issue by analyzing the proportion of CD4^+^ T cells that represent Tregs in SSc patients and healthy control individuals^6^,^7^,^8^,^9^,^10^,^11^. However, these studies have reported diverse results. Some studies found that the ratio of Tregs to CD4^+^ T cells is significantly decreased^6^,^8^,^10^ or increased^7^ in SSc patients compared to that in control individuals, while the other found no significant difference between the two groups^9^,^11^. Moreover, the source of these inconsistencies is yet unexplored.

Meta-analysis is a powerful method to synthesize information from various studies and generate conclusions based on the analyzed studies. In addition, analysis of the heterogeneity of the enrolled studies in meta-analyses helps to identify sources of inconsistencies. In this study, we have performed a meta-analysis of published literature to understand whether or not changes in the ratio of circulating Tregs to CD4^+^ T cells are indeed relevant in the case of SSc.

## Methods

### Literature search

Relevant studies were identified in the databases of Pubmed, Embase, and ISI web of knowledge. To retrieve all relevant publications related to Tregs in SSc patients, database searches were performed using the following keywords: “regulatory T cell”, “Treg”, “CD4^+^ CD127 T cell”, “CD4^+^CD25^+^ T cell”, “CD4^+^ CD25^high^ T cell”, “CD4^+^ CD25^bright^ T cell”, “CD4^+^CD25^+^ Foxp3^+^ T cell”, “CD4^+^ CD25^high^ Foxp3^+^ T cell”, “CD4^+^ CD25^bright^ Foxp3^+^ T cell”, combined “scleroderma” and “systemic sclerosis”. Review articles were filtered out. No limits were placed on ethnicity or geographic region and all documents were updated to Feb 2016. Additional relevant references cited in searched articles were also selected.

### Eligibility criteria

Studies meeting the following criteria were eligible for inclusion in the meta-analysis: (1) designed as case-control study; (2) assessed the changes in peripheral blood Tregs to CD4^+^ T cell proportion in patients with SSc; (3) provided sufficient data, including mean and standard deviation of Treg/CD4^+^ T cell from case and control, to calculate the efficient size. No restriction was placed on the language in which the study was published. In the case of overlapping studies, only the study with the largest sample size was included in our analysis. Studies based on animals or cell culture, case reports, and conference abstracts without subsequent publications were excluded from the analysis.

### Data extraction

Data was extracted from all selected studies by 2 independent investigators. Inter-researcher disagreements were resolved by consensus. The following data were collected from each selected study: first author’s name; publication year; country from which the participants were recruited; diagnosis criteria; Treg definition; treatment of the enrolled participants, and study results. Study quality was assessed using the Newcastle-Ottawa Scale (NOS). Authors of the identified studies were contacted via e-mail if further details were needed.

### Statistical analysis

Statistical analysis was performed using Comprehensive Meta Analysis Version 2.0 software (Englewood, USA). The *I*^*2*^-statistic was also used to evaluate heterogeneity, with *I*^*2*^ < 25% considered as low heterogeneity, 25–50% as moderate, and >50% as high degree of inconsistency. Finally, the overall or pooled weighted mean difference and its 95% CI was obtained by a random-effects or a fixed-effects model in the presence (*I*^*2*^ > 50%) or absence (*I*^*2*^ ≤ 50%) of heterogeneity respectively. The subgroup analysis and publication bias analysis were also performed.

## Results

### Literature search

**Figure 1:**
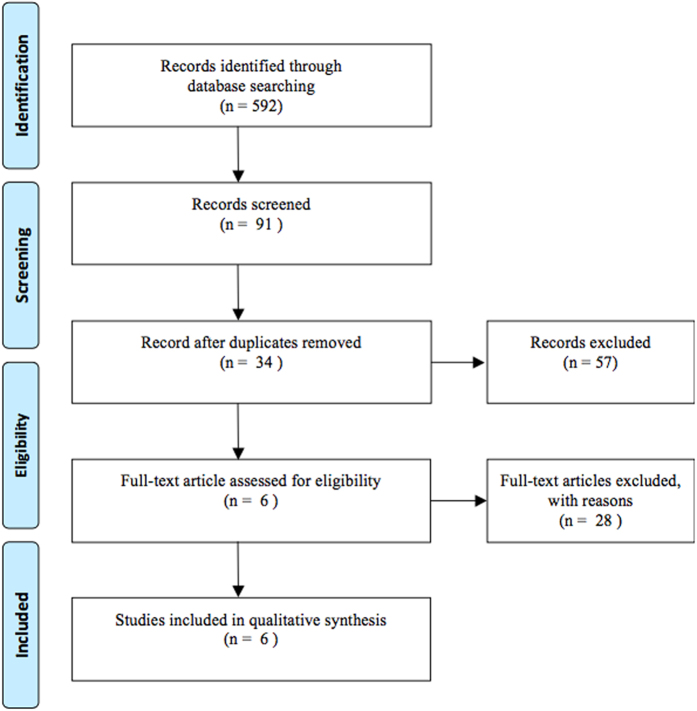
Flow chart of studies included in the meta-analysis.

Electronic and manual searches yielded 592 potentially eligible articles. A flow chart of the screening protocol applied to these the articles for meta-analysis is shown in Fig. 1 Five hundred and one articles were excluded by screening the titles and abstracts. Of the remainder, 57 duplicate articles were excluded. An additional 28 articles were excluded for the following reasons: one study wrongly gated the Treg population; two studies were not designed to detect changes of Treg in controls; three studies focused on changes in the Treg numbers in samples other than peripheral blood mononuclear cell; eleven did not provide sufficient data, and a further eleven were unrelated. A total of 6 eligible studies were finally included in the meta-analysis^6^,^7^,^8^,^9^,^10^,^11^.

### Study characteristics

The characteristics of the 6 studies are summarized in Table 1. A total of 135 SSc patients and 112 control individuals were involved in these studies. With regards to the geographic location of the studies, 3 were carried out in China^7^,^8^,^9^, 1 in Hungary^6^, 1 in France^10^ and 1 in Japan^11^. Assessment using NOS indicated that the studies were of medium quality (all of the NOS score ≥ 6, Table 1).

### Meta-analysis of the changes of peripheral blood Tregs in SSc patients compared to controls

**Figure 2:**
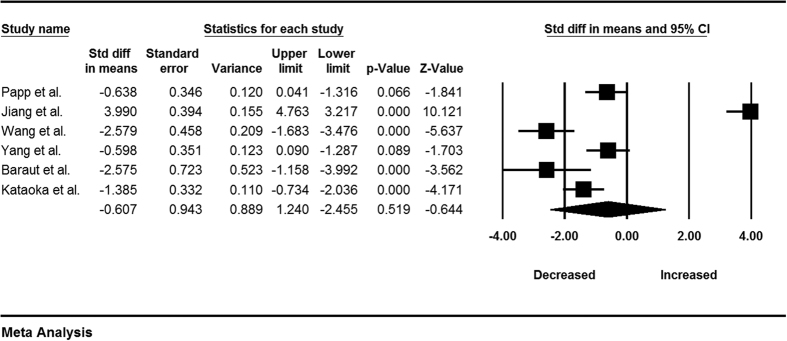
Forest plot of the changes of peripheral blood Tregs to CD4^+^ T cell in SSc patients compare with controls.

In the 6 studies we selected, 4 reported significantly decreased proportions of Tregs in SSc patients compared to control individuals^6^,^8^,^10^,^11^, 1 found decreased proportions of Tregs in SSc patients but not significantly different between the two groups^9^, and 1 study showed increased proportions of Tregs^7^. Results of the meta-analysis showed high degree of heterogeneity (*I*^*2*^ = 96.98), and hence, a random-effect model was applied for further analysis. In the overall analysis, the ratio of Tregs to CD4^+^ T cells in SSc patients was lower than controls, but the difference was not statistically significant (−0.61 ± 0.94, P = 0.52). The forest plots for these analyses are shown in Fig. 2. The observed heterogeneity in these data may originate from differences in the countries in which the analyses were performed, cell surface markers used for enumeration of Tregs, diagnosis criteria, and treatment strategies.

### Subgroup analyses and publication bias

Subgroup analyses were performed on the aforementioned factors to explore the sources of heterogeneity in the data. Subgroup analysis could be performed only if each category of the subgroup contained more than one article. As a result, differences in the countries from which the participants originated and treatment strategies of the recruited patients that did not fulfill this requirement were excluded from the subgroup analysis. High heterogeneities were also found in the other two subgroups, namely cell surface marker and diagnosis criteria (*I*^*2*^ = 97.57 and *I*^*2*^ = 97.78), and the random-effect model was applied for further analysis.

The Treg definition that was only applied once among recruited studies could not be considered as a group. Therefore, data from these studies were dismissed from the subgroup analysis. We found that Tregs were defined either as as CD25^+^ (CD25 normal expression) or CD25^high^ (CD25 high expression). In the recruited articles, 3 used CD25^+^ and 2 used CD25^high^ to define Tregs. We found that the ratio of CD4^+^CD25^+^ Foxp3^+^ Tregs to CD4^+^ T cells was elevated while the ratio of CD4^+^ CD25^high^ Foxp3^+^ T cells to CD4^+^ T cells was diminished, but this was not statistically significant (0.11 ± 1.80 vs. −1.59 ± 2.22, P = 0.66).

Four diagnostic criteria^12^,^13^,^14^,^15^ were commonly used in most studies related to SSc. Of these, 3 criteria are reported in the selected studies. Two of the recruited articles did not report diagnosis criteria (Table 1). The diagnosis criteria that were only applied once among the recruited studies could not be considered as a group, and data from these articles were dismissed from subgroup analysis. Subgroup analysis showed that studies that did not state their diagnostic criteria reported a higher ratio of Tregs to CD4^+^ T cells than the studies that used diagnosis criteria established by ACR in 1980^12^. However, these differences were also not statistically significant (0.81 ± 1.72 vs. −0.99 ± 1.69, P = 0.93).

Egger linear regression analysis and Begg rank correlation test were used to evaluate publication bias, if any. No bias was found (Intercept = −4.91, P = 0.74).

### Additional meta-analysis of the changes of peripheral blood Tregs in SSc

The immune system of SSc patients is expected to change after treatment; however, the underlying mechanism has not been fully elucidated. As an important factor of cell-mediated immunity, establishing the changes of Tregs as a proportion of CD4^+^ T cells before and after treatment in SSc patients may help understand how current therapy strategies act on the immune system. We therefore re-checked the 592 potentially eligible articles and found 2 additional studies^16^,^17^ that focused on the above aspect (Table 1). Meta-analysis revealed that medium heterogeneity was found among these studies (*I*^*2*^ = 34.72 for treatment and *I*^*2*^ = 43.54 for disease activity) and a fixed-effect model was applied for subsequent analyses. The ratio of Tregs to CD4^+^ T cell in SSc patients before treatment was significantly lower than that after treatment (−0.92 ± 0.29, P < 0.01). A comparison of data from active and stable SSc patients showed that patients with active SSc had higher ratio of Tregs to CD4^+^ T cells than stable patients. However, this difference was not statistically significant (−0.13 ± 0.25, P = 0.61).

## Discussion

Among the studies selected for this meta-analysis, 4 reported significantly decreased proportions of Tregs in SSc patients compared to control individuals^6^,^8^,^10^,^11^, 1 found decreased proportions of Tregs in SSc patients but not significantly different between the two groups^9^, and 1 study showed increased proportions of Tregs^7^. For this meta-analysis, we collated information from each study and concluded that the ratio of Tregs to CD4^+^ T cells in SSc patients tends to be lower than that in controls, but this difference was not statistically significant (Fig. 2).

Generally, Tregs play an anti-inflammatory role, and their interaction with pro-inflammatory effector T cells (including Th cells, NKT cells, and others) results in a network of immune regulation. A decrease in the number of Treg cells or an increase in the number of the other effector T cell can both result in a reduction in the ratio of Tregs to CD4^+^ T cells. This reduced ratio suggests that Tregs might play a less prominent immunosuppressive role in the immune system. Our analyses suggest that the decreased ratio of Tregs to CD4^+^ T cells in SSc maybe an important source of the Tregs dysfunction. Since high heterogeneity was found in this analysis (*I*^*2*^ = 96.98), we performed subgroup analysis to explore the reason for the heterogeneity.

It may be expected that treatment might influent the immune system of autoimmune diseases. We therefore performed the subgroup analysis with the above factor in mind. We further analyzed the ratio of Tregs to CD4^+^ T cells in patients before and after treatment. Interestingly, an elevated proportion of Tregs was found in SSc patients after treatment. However, we could not draw a definitive conclusion in this regard for three reasons. First, the statistical power of the analysis was not enough since only 2 studies were used for this part of the analysis. Second, the types and dosages of drugs used in the treatment of SSc might have different effects on the immune system of patients, which might influent the result. Third, our additional meta-analysis showed that active SSc patients have higher proportions of Tregs than stable ones, which suggests that disease activity might be one of the factors that influent the ratio of Tregs to CD4^+^ T cells. Since the disease activity of the SSc patients was not reported in the two studies focusing on treatment status, we cannot rule out the possibility that might result in the phenomenon we observed.

The diagnostic criteria for SSc established in 1980^12^ were not sensitive enough to detect early and limited SSc. The addition of wide field nailfold microscopy and SSc related autoantibodies^13^,^14^ resulted in a significant improvement of the sensitivity of diagnosis of SSc. Importantly, studies that applied different diagnostic criteria might recruit different SSc patients. Our subgroup analysis showed the studies that did not report their diagnosis criteria estimated a higher ratio of Tregs in CD4^+^ T cells than the studies using diagnosis criteria established by ACR in 1980. Since the former did not report their diagnosis criteria, we cannot explore the differences between these two groups in greater depth. In addition, the treatment status of the SSc patients recruited in this subgroup analysis also differed and included treated, untreated, and mixed patient cohorts, making further investigation more difficult.

To the best of our knowledge, there are at least 7 kinds of definitions used for Tregs based on different cell surface markers, namely CD4^+^CD25^+^, CD4^+^CD25^+^CD127^−^, CD4^+^CD25^high^, CD4^dim^CD25^high^, CD4^+^CD25^+^FoxP3^+^, CD25^+^FoxP3^+^CD127^−^ and CD4^+^CD25^high^FoxP3^+^. In most of the analyzed studies, Tregs were defined as CD4^+^CD25^+^FoxP3^+^ and CD4^+^CD25^high^FoxP3^+^. Obviously, the major difference between these two markers is the expression level of CD25. The expression of CD25 is not, however, restricted to Tregs as it has also been detected in active T effector lymphocytes^18^. Some studies have reported that Tregs defined with high expression level of CD25 exhibited a powerful regulatory function^19^. To exclude the possibility that different expression levels of CD25, and in turn, the different definitions of Tregs used in different studies might be responsible for the observed heterogeneity, we performed the subgroup analysis of the cell surface marker of Tregs. In our meta-analysis, no significant difference between the change in the ratio of Tregs to CD4^+^ T cells was found between studies using CD4^+^CD25^+^FoxP3^+^ and CD4^+^CD25^high^FoxP3^+^ for defining Tregs. Based on the data analyzed in this study, no heterogeneity was found to be introduced by different gating strategies using different express levels of CD25 on T cells. Since studies that used other definition of Tregs were less than one or not used in our meta-analysis, we are unable to conclude whether different definitions of Tregs bring in heterogeneity or not.

Some limitations in this meta-analysis need to be noted. According to the current result, we cannot rule out that cell surface markers used for enumeration of Tregs, diagnosis criteria, treatment strategies and others factors in the studies may have an effect on the heterogeneity. Since the number of articles selected for the analysis were less than 10, it would not be appropriate to perform regression analysis for these parameters. Secondly, some studies were excluded from this analysis as their results were represented as median of the percentage, which cannot be used for further calculations. Dismissing these studies is expected to weaken the strength of the results obtained from this In conclusion, the ratio of Tregs to CD4^+^ T cells in SSc patients tends to be lower than in healthy control individuals. In the future, more studies will no doubt enhance the current analysis. In addition, new studies should be designed carefully, with larger sample sizes as well as a careful monitoring of treatment status, diagnosis criteria and cell surface markers.

## Additional Information

**How to cite this article**: Deng, C. *et al*. Meta-analysis of the changes of peripheral blood regulatory T cell to CD4^^+^^ T cell ratio in patients with systemic sclerosis. *Sci. Rep.* **7**, 43532; doi: 10.1038/srep43532 (2017).

**Publisher's note:** Springer Nature remains neutral with regard to jurisdictional claims in published maps and institutional affiliations.

## Figures and Tables

**Table 1:** Characteristics of studies included in the meta-analysis.

Meta-analysis	Year	Author	Region	Diagnosis criteria	Treatment Status	Treg definition	Case (Number)	Control (Number)	Tregs in (Casemean ± SD, %)	Tregs in Control (mean ± SD, %)	NOS score
Overall analysis											
	2011	Papp *et al*.^6^	Hungary	1988 criteria	Some patients treated	CD4^+^CD25^high^FoxP3^+^	21	15	5.03 ± 2.29	6.21 ± 0.91	8
	2014	Jiang *et al*.^7^	China	Not report	Not report	CD4^+^CD25^+^FoxP3^+^	53	27	3.04 ± 0.19	2.24 ± 0.22	6
	2014	Wang *et al*.^8^	China	2013 ACR criteria	All patietns treated	CD4^+^CD25^+^FoxP3^+^	18	17	6.20 ± 1.80	11.10 ± 2.00	8
	2014	Yang *et al*.^9^	China	1980 ACR criteria	All patietns treated	CD4^+^CD25^+^CD127^−^	13	24	6.25 ± 1.22	7.14 ± 1.61	8
	2014	Baraut *et al*.^10^	France	Not report	All patietns treated	CD4^+^CD25^high^FoxP3^+^	7	7	2.00 ± 0.50	4.20 ± 1.10	6
	2015	Kataoka *et al*.^11^	Japan	1980 ACR criteria	All patietns untreated	CD4^+^CD25^+^FoxP3^+^	23	22	2.73 ± 1.46	4.80 ± 1.53	7
Treatment							Untreat (Number)	Treated (Number)			
	2012	Papp *et al*.^16^	Hungary	1988 criteria	photopheresis	CD4^+^CD25^+^FoxP3^+^	19	19	4.97 ± 1.23	5.91 ± 1.36	6
	2014	Baraut *et al*.^10^	France	Not report	autologous hematopoietic SCT	CD4^+^CD25^high^FoxP3^+^	7	7	2.00 ± 0.50	4.10 ± 1.80	6
Disease activity							Active (Number)	Stable (Number)			
	2005	Matsui *et al*.^17^	Japan	2001 criteria	All patietns treated	CD4^+^CD25^+^	27	9	2.70 ± 1.40	2.00 ± 1.10	7
	2014	Yang *et al*.^9^	China	1980 ACR criteria	All patietns treated	CD4^+^CD25^+^CD127^−^	13	32	6.25 ± 1.22	6.47 ± 1.49	8
